# Long-Term Outcomes of Modified Expansive Open-Door Laminoplasty Combined with Short-Level Anterior Cervical Fusion in Multilevel Cervical Spondylotic Myelopathy

**DOI:** 10.3390/medicina60122057

**Published:** 2024-12-13

**Authors:** Szu-Wei Chen, Kuang-Ting Yeh, Cheng-Huan Peng, Chia-Ming Chang, Hao-Wen Chen, Tzai-Chiu Yu, Ing-Ho Chen, Jen-Hung Wang, Wan-Ting Yang, Wen-Tien Wu

**Affiliations:** 1 School of Medicine, Tzu Chi University, Hualien 970, Taiwan; 108311145@gms.tcu.edu.tw (S.-W.C.); micrograft@tzuchi.com.tw (K.-T.Y.); peng0913@tzuchi.com.tw (C.-H.P.); feyu@tzuchi.com.tw (T.-C.Y.); ihchen@tzuchi.com.tw (I.-H.C.); 2 Department of Orthopedics, Hualien Tzu Chi Hospital, Buddhist Tzu Chi Medical Foundation, Hualien 970, Taiwan; fresheric@tzuchi.com.tw (C.-M.C.); charliechen728@tzuchi.com.tw (H.-W.C.); 3 Graduate Institute of Clinical Pharmacy, Tzu Chi University, Hualien 970, Taiwan; 4 Institute of Medical Sciences, Tzu Chi University, Hualien 970, Taiwan; 5 Department of Medical Education, Hualien Tzu Chi Hospital, Buddhist Tzu Chi Medical Foundation, Hualien 970, Taiwan; 6 Department of Medical Research, Hualien Tzu Chi Hospital, Buddhist Tzu Chi Medical Foundation, Hualien 970, Taiwan; paulwang@tzuchi.com.tw; 7 Department of Nursing, Meiho University, Pingtung 912, Taiwan; x00012168@meiho.edu.tw

**Keywords:** cervical spondylotic myelopathy, laminoplasty, anterior cervical fusion, spinal cord compression, adjacent segment degeneration

## Abstract

*Background and Objectives*: Multilevel cervical spondylotic myelopathy (MCSM) presents complex challenges for surgical management, particularly in patients with kyphosis or significant anterior pathology. This study aimed to assess the long-term efficacy of modified expansive open-door laminoplasty (MEOLP) combined with short-level anterior cervical fusion (ACF) in providing decompression, preserving alignment, and maintaining range of motion (ROM) over a nine-year follow-up. *Materials and Methods*: A retrospective analysis was conducted on 124 MCSM patients treated with MEOLP combined with ACF between 2011 and 2015. MEOLP, a muscle-sparing posterior approach, was combined with ACF to correct sagittal misalignment and address anterior compression. Key outcome measures included the Pavlov ratio, C2–C7 angle, Japanese Orthopedic Association (JOA) score, and Visual Analog Scale (VAS) for neck pain. Patients were monitored for adjacent segment degeneration (ASD) and other postoperative changes over the long-term follow-up. *Results:* At nine years post-surgery, patients demonstrated significant improvements in decompression and cervical alignment. The mean C2–C7 angle increased, reflecting enhanced lordotic curvature, while the Pavlov ratio showed maintained canal expansion. JOA scores improved significantly, indicating reduced myelopathy symptoms, and VAS scores for neck pain decreased, reflecting symptom relief. Despite these positive outcomes, ASD was noted, especially in patients with reduced preoperative disk height, highlighting the need for strategies to mitigate degeneration at adjacent segments. *Conclusions*: MEOLP combined with short-level ACF is a viable and durable option for managing complex MCSM cases, offering effective decompression, alignment correction, and ROM preservation. The limitations of this study, including its retrospective, single-center design and the lack of quality-of-life assessments, underscore the need for future multi-center studies with broader outcome measures. These findings support MEOLP with ACF as an alternative approach in cases where traditional laminoplasty may be insufficient.

## 1. Introduction

Cervical spondylotic myelopathy (CSM) is a progressively debilitating neurological condition that arises from degenerative spinal changes that cause compression of the spinal cord and surrounding structures [1]. Disk herniation, osteophyte formation, and ligament thickening can gradually narrow the spinal canal, often compressing the spinal cord at multiple levels, resulting in multilevel cervical spondylotic myelopathy (MCSM) [2]. These conditions manifest as neck pain, cervical radiculopathy, sensory deficits, and other spinal cord syndromes, progressively diminishing patient quality of life [3,4,5]. Conservative treatments, such as neck immobilization, anti-inflammatory medications, physical therapy, and lifestyle modifications, may offer initial symptom relief but are often insufficient for patients with severe, persistent cord compression or MCSM symptoms [6,7]. For these patients, surgical intervention is the preferred approach to alleviate spinal cord compression, improve symptoms, and prevent further deterioration [8,9].

Surgical approaches for CSM generally include anterior, posterior, or combined techniques, depending on cervical alignment, the location of cord compression, the number of affected levels, and the presence of instability [10,11]. Anterior cervical decompression fusion (ACF)—which includes anterior cervical discectomy with fusion and anterior cervical corpectomy with fusion—is favored in cases with anterior pathology (e.g., disks, osteophytes), cervical kyphosis, or limited segment involvement [12]. By contrast, posterior approaches, including laminectomy, laminectomy with fusion, and laminoplasty, are typically used for extensive pathologies such as MCSM [13,14]. Expansive open-door laminoplasty (EOLP), initially introduced by Kiyoshi et al. [15], preserves stability, maintains spinal mobility, and avoids fusion-related complications compared with laminectomy [16]. However, EOLP can lead to complications such as axial neck pain, C5 palsy, and lamina arch reclosure. To address these challenges, we developed modified expansive open-door laminoplasty (MEOLP), a minimally invasive, muscle-sparing approach that preserves posterior elements and minimizes facet joint damage [17]. In our prior studies, the average patient who had undergone MEOLP had an 82.3% Japanese Orthopedic Association (JOA) recovery rate, 85% of their cervical range of motion (ROM) preserved, reduced neck pain, and no presentation of C5 palsy or lamina reclosure at midterm follow-up [18].

Cervical alignment alleviates spinal cord dysfunction caused by direct compression, repetitive flexion-extension injuries, or vascular compromise, improving surgical outcomes and quality of life for patients with MCSM [19]. Laminoplasty alone may result in suboptimal outcomes for patients with both MCSM and kyphotic deformities, which limit posterior cord drift post-decompression [20,21]. Studies have shown that laminoplasty can also exacerbate preexisting kyphosis and contribute to segmental instability [22,23]. A combined approach involving MEOLP and short-level ACF can balance stability and mobility preservation and address localized spinal concerns for patients with MCSM presenting with segmental instability, kyphosis, or significant anterior pathology.

Our previous findings indicated favorable short- and medium-term outcomes for MCSM patients treated with both MEOLP and short-level ACF [24,25]. However, the literature on the long-term outcomes of this combined approach is limited. To fill this gap, the present study investigated 9-year follow-up functional and radiographic results for patients with MCSM who underwent MEOLP with short-level ACF.

## 2. Materials and Methods

Our retrospective study was conducted with approval from the Ethics Committee of Hualien Tzu Chi Hospital (IRB 112-130-B). The inclusion criteria of the patients included those who underwent MEOLP with ACF between 2011 and 2015, and each had a minimum follow-up duration of 9 years. Patients were included if they had (1) C3–7 spinal stenosis with spinal cord compression; (2) diagnosis as MCSM based on the symptoms, results of physical examination, and cervical MRI by Dr. Wu or Dr. Yeh; (3) a combination of one- or two-level local kyphosis (Cobb angle < 0 degree) [26], segmental instability (horizontal displacement (≥0.3) and angular displacement on dynamic X-ray films) [27], or major anterior pathology [25]; (4) received C3–6 MEOLP with one- or two-level ACF between 2011 and 2015 [24]; and (5) a minimum follow-up duration of 9 years. Patients were excluded if they had confounding systemic conditions that could influence functional outcomes, such as neurodegenerative diseases (like Parkinson’s disease), rheumatoid arthritis, spondyloarthritis, significant cardiovascular diseases, poorly managed diabetes, or any urological disorders that could interfere with JOA scoring.

To minimize inter-operator variability, all surgical procedures were conducted by the same surgeon (Dr. Wu), using a standardized protocol established during a pilot phase of the study. Instrumentation and techniques were rigorously monitored to maintain uniformity across the cohort [24]. The surgical procedures were introduced as below. The combined MEOLP and ACF procedure was performed to address both posterior and anterior pathologies of the cervical spine. MEOLP is a modified version of EOLP that minimizes muscle disruption, preserves cervical alignment, and maintains spinal mobility by limiting facet joint involvement. During MEOLP, paraspinal muscles were unilaterally dissected, and the spinous processes from C3 to C6 on the hinged side were removed to allow posterior access. To prevent excessive facet joint damage, lateral gutters were created near the spinous processes, thereby preserving joint integrity. A partial laminectomy at C7 was then performed, and the elevated laminae from C3 to C6 were secured using titanium miniplates and screws to ensure stability. Finally, the semispinalis cervicis muscle was carefully sutured back to its original position to maintain muscle attachment and further reduce postoperative complications. ACF was only performed when the anterior pathology significantly contributed to the patient’s condition and required targeted decompression, such as in cases caused by disk herniation, osteophytes, or other anterior structures that could not be fully addressed by posterior MEOLP alone. By combining MEOLP with ACF, the procedure addressed both posterior and anterior elements of compression, which was particularly useful for cases with multilevel involvement and mixed anterior–posterior pathology.

Following surgery, patient recovery was closely monitored for complications. Patients were initially fitted with a cervical collar, which they were advised to wear continuously for the first three postoperative months to support healing and reduce strain on cervical structures. After the initial 3-month recovery period, patients were encouraged to participate in a structured rehabilitation program emphasizing neck extension exercises to regain and preserve cervical mobility. Clinical and radiographic evaluations were conducted every 3 months during the first postoperative year and annually thereafter, allowing for comprehensive longitudinal follow-up.

To evaluate the effectiveness of MEOLP combined with ACF, a wide range of demographic, radiographic, and functional parameters were collected both preoperatively and postoperatively. Each patient underwent anteroposterior and neutral lateral cervical spine X-rays to evaluate static alignment and dynamic MRI imaging to assess functional mobility and soft tissue status.

### 2.1. Pavlov Ratio

The Pavlov ratio [28], or Torg–Pavlov ratio, represents spinal canal diameter relative to vertebral body width and is commonly used to detect cervical spinal stenosis. The Pavlov ratio was measured at the most affected cervical level minus two (MACL-2), as determined by preoperative and postoperative MRI scans. This approach was chosen to ensure the measurement corresponded to the region of greatest stenosis severity, providing a more precise evaluation of spinal canal narrowing. A preoperative Pavlov ratio below 0.82 is commonly associated with significant stenosis, which was prevalent among our patient group. Radiographic changes in the Pavlov ratio were tracked over the follow-up period to evaluate the extent of spinal decompression achieved using MEOLP with ACF.

### 2.2. C2–C7 Angle (Cervical Lordosis)

The C2–C7 angle, a measure of cervical lordosis, was used to assess sagittal alignment of the cervical spine, determined by drawing lines along the inferior endplate of C2 and superior endplate of C7 on a neutral lateral X-ray [29]. The angle between these two lines represents the overall curvature of the cervical spine, with positive values indicating lordosis and negative values indicating kyphosis. Preoperative kyphosis, a common concern for patients with MCSM, often hinders laminoplasty success when performed without additional surgical intervention. Patient C2–C7 angles were tracked over the 9-year follow-up period to determine long-term cervical alignment stability and assess surgical effectiveness in maintaining or improving lordotic curvature.

### 2.3. C2–C7 Sagittal Vertical Axis (CSVA)

The CSVA is a marker of sagittal balance and functional alignment measured according to the horizontal distance from the center of the C2 vertebra to the posterosuperior corner of C7 on a neutral lateral radiograph [29]. Positive CSVA values indicate anterior translation of the cervical spine, which is often linked to instability and compensatory adjustments in cervical alignment. The present study assessed changes in the CSVA from preoperative to long-term postoperative stages to understand sagittal alignment stability and its influence on functional outcomes. An increase in the CSVA over time would suggest increased cervical strain and a greater risk of adjacent segment disease (ASD).

### 2.4. C7 Slope

The C7 slope, another key measure of cervical alignment, is the angle formed between the superior endplate of C7 and a horizontal reference line [29]. Positive values indicate lordosis, whereas negative values indicate kyphosis. The C7 slope is crucial for maintaining head balance and overall cervical alignment, causing maladaptive postural changes and neck strain when altered, which are major concerns for patients undergoing laminoplasty. The present study monitored preoperative and postoperative C7 slope changes to capture shifts in cervical orientation that could affect long-term outcomes.

### 2.5. Adjacent-Level Disk Heights

The heights of disks adjacent to the fused spinal segments were measured to assess their risk of degeneration. Upper disk height (UDH) and lower disk height (LDH) were recorded at the proximal and distal adjacent levels, respectively, using anteroposterior X-ray imaging. Disk height reduction at levels adjacent to the fused segments can signify progressive degeneration or compensatory adjustments due to reduced motion of the fused segments. Both preoperative and postoperative UDH and LDH were compared to identify surgery-related changes.

### 2.6. ROM

Overall ROM was calculated by measuring the difference in angle between the lower endplate of C2 and the upper endplate of C7 on dynamic lateral X-rays (flexion and extension views). Additionally, proximal (UROM) and distal (LROM) ROM at levels adjacent to fused segments were recorded to monitor compensatory mobility. Although some ROM reduction is anticipated following fusion, excessive loss could affect functional outcomes. ROM data were used to examine the degree of mobility preservation and any long-term surgical effects on adjacent segments.

The functional outcomes assessed were neurological function, which was measured using the JOA score, and pain perception, which was measured using the Visual Analog Scale (VAS) for neck pain.

#### 2.6.1. JOA Score

The JOA score was used to assess motor, sensory, and bladder components of neurological function out of a maximum of 17 points, with higher values indicating better function. The difference between preoperative and postoperative JOA scores was calculated to assess improvement and recovery rate using the following formula: (postoperative JOA score − preoperative JOA score)/(17 − preoperative JOA score) × 100%.

#### 2.6.2. Neck VAS

VAS scores for neck pain, ranging from 0 (no pain) to 10 (worst imaginable pain), were used to assess subjective pain levels, with changes over time used to measure pain relief achieved through surgical intervention.

Statistical analyses were performed using IBM SPSS Statistics software for Windows 10 (version 23.0; IBM, Armonk, NY, USA). Kolmogorov–Smirnov tests for normality were conducted. An independent *t*-test and Wilcoxon rank-sum test were used to identify significant differences between men and women in clinical and radiographic characteristics for normally and nonnormally distributed variables, respectively. A chi-squared test was used to evaluate the association between categorical variables, and the relationship between radiographic or functional parameters and long-term outcomes was examined using multiple logistic regression analysis. Data are presented as the mean ± standard deviation for continuous variables or as β with a 95% confidence interval (CI) for regression coefficients. A *p*-value of less than 0.05 indicated statistical significance. Using G*Power software (version 3.1.9.2), we determined a requisite minimum sample size of 120 on the basis of an effect size of 0.12, an α of 0.05, a power (1 − β) of 0.80, and a number of predictors of 22 in a multiple linear regression of cervical spine sagittal alignment with respect to clinical and radiographic parameters.

## 3. Results

The present study followed 124 patients with MCSM who underwent MEOLP combined with short-level ACF for a minimum of 9 years post-operation. The cohort consisted of 64 male and 60 female patients, and the presence of myelomalacia on preoperative MRI was identified in 34 individuals (22 male and 12 female). Baseline demographic data and key radiographic and functional measures (Table 1) provided a comprehensive starting point for evaluating the long-term effects of MEOLP combined with ACF. There were 84 patients receiving single-level ACF and the other 40 patients undergoing two-level ACF. The distribution of treated levels was as follows: 44 patients received ACF at the C4–C5 level, 20 patients at C5–C6, and 20 patients at C3–C4. Among those undergoing two-level ACF, 24 patients were treated at C3–C5, and 16 patients at C4–C6.

Table 1 presents an in-depth look at the demographic and clinical characteristics of the study population, including differences between male and female participants at structural and functional baselines. The mean age for the entire cohort was 56.71 ± 11.75 years, with a slight difference between male (55 ± 11.89) and female (58.53 ± 11.71) participants. Preoperative myelomalacia was more prevalent in male (68.8%) than female (40.0%) participants, although this difference was not statistically significant (*p* = 0.108). Key radiographic indicators, including the Pavlov ratio, C2–C7 angle, and C7 slope, were used to assess cervical spinal alignment and stability. The Pavlov ratio did not significantly differ (*p* = 0.586) between male (0.71 ± 0.05) and female (0.72 ± 0.04) participants. The C2–C7 angle had a mean value of −8.88° ± 10.02° in male participants and −8.53° ± 11.73° in female participants, indicating mild kyphosis. The C7 slope was measured at 16.19 ± 6.78 in male participants and 13.80 ± 4.21 in female participants, without significant differences between them. UDH and LDH proximal and distal to the fusion site were recorded to assess changes in adjacent segment integrity. Male participants had an average UDH of 6.47 ± 0.84 mm, slightly but significantly (*p* = 0.049) higher than female participants (5.94 ± 0.53 mm). LDH values were comparable between both groups (6.33 ± 0.83 mm in male participants and 5.90 ± 1.01 mm in female participants, *p* = 0.208). Baseline ROM values indicated substantial movement in the cervical spine, with an overall ROM of 46.60° ± 13.61°. Notably, male participants had lower baseline JOA scores (10.56 ± 1.26) compared with female participants (11.40 ± 0.74), a significant difference (*p* = 0.033), while neck pain, assessed using VAS scores, was similar between groups.

Radiographic parameters showed notable postoperative changes that remained apparent at the 9-year follow-up, as shown in Table 2. Pavlov ratios, C2–C7 angles, CSVAs, and C7 slopes reflected the structural effects of the combined MEOLP and ACF procedure over time. The mean Pavlov ratio at the MACL-2 level increased significantly from 0.72 ± 0.05 preoperatively to 0.90 ± 0.04 immediately after surgery, a difference of 0.19 ± 0.05 (*p* < 0.001), indicating improved spinal canal diameter. At the 9-year follow-up, the mean Pavlov ratio remained relatively high at 0.87 ± 0.04, although this represents a slight decrease (difference of 0.16 ± 0.06; *p* < 0.001) from the immediate postoperative measure. The C2–C7 angle indicated a marked postoperative improvement in cervical lordosis. Preoperatively, the angle averaged −8.71° ± 10.70°, indicative of mild kyphosis, and improved to −15.23° ± 9.49° postoperatively (difference of −6.52° ± 8.68°, *p* < 0.001), reflecting an increase in lordotic curvature that was largely preserved at the 9-year follow-up (−14.90° ± 9.33°). The C7 slope and CSVA also exhibited significant postoperative changes, which were largely sustained over the long term. The C7 slope increased from a preoperative mean value of 15.03 ± 5.72 to 20.06 ± 7.07 immediately after surgery, with a further increase to 22.45 ± 7.21 at 9 years (*p* < 0.001). Similarly, the CSVA increased from 15.68 ± 9.90 preoperatively to 24.04 ± 11.11 immediately after surgery and then to 25.07 ± 12.90 at the 9-year follow-up (*p* < 0.001). UDH decreased from 6.02 ± 0.74 mm postoperatively to 5.82 ± 0.79 mm at 9 years (*p* = 0.003), and LDH exhibited a similar decrease from 6.12 ± 0.93 mm to 5.54 ± 1.10 mm over the same period (*p* < 0.001). These decreases highlight the gradual changes in adjacent segment disk heights, potentially indicating ASD over time. ROM decreased substantially from a preoperative mean of 46.60° ± 13.61° to 20.80° ± 6.88° immediately after surgery, a difference of −25.80° ± 13.12° (*p* < 0.001). At the 9-year follow-up, total ROM was 19.83° ± 8.70°, indicating an anticipated long-term reduction due to fusion. Despite the decline in ROM, the preservation rates of UROM and LROM at adjacent segments remained relatively stable, with 78% of UROM and 47% of LROM maintained after 9 years.

Functional outcomes at the 9-year follow-up mark showed notable improvement (Table 2). The mean JOA score improved from 10.97 ± 1.11 preoperatively to 15.52 ± 0.68 postoperatively, with an increase to 16.10 ± 0.94 at 9 years (*p* < 0.001). Similarly, the neck pain VAS score decreased from 6.52 ± 0.81 preoperatively to 3.00 ± 0.82 postoperatively, with a slight increase to 3.16 ± 1.34 at 9 years (*p* < 0.001). Table 3 presents a logistic regression analysis identifying factors associated with JOA and neck pain VAS score changes. The following postoperative changes were predictive of long-term neck pain: (1) UDH changes: A greater decrease in UDH was significantly associated with increased neck pain at 9 years, with a β value of 1.46 and a CI of (0.26, 2.66), *p* = 0.020; (2) ROM and LROM changes: Reductions in overall ROM and LROM were also linked to greater neck pain, with a β of 0.10 (CI: 0.03, 0.17; *p* = 0.009) for ROM and β of −0.11 (CI: −0.22, −0.001; *p* = 0.049) for LROM. These findings suggest that changes in adjacent-level disk height and ROM may be critical factors in predicting long-term neck pain following MEOLP with ACF. Although age, sex, and presence of myelomalacia were also analyzed, these factors had no significant association with JOA or neck pain VAS scores.

Overall, the combined MEOLP and ACF surgical technique resulted in durable improvements to cervical alignment, canal dimensions, and functional outcomes, with significant reductions in neck pain and preservation of UROM over the long term. These findings underscore the importance of targeted assessment and postoperative monitoring, particularly for adjacent disk heights and ROM, to optimize patient outcomes and mitigate long-term neck pain risk.

## 4. Discussion

The present study included a comprehensive 9-year follow-up on outcomes of MEOLP combined with short-level ACF in patients with MCSM. Our results indicated that MEOLP with ACF effectively addressed multilevel cervical stenosis by providing sufficient decompression, maintaining cervical alignment, and improving functional outcomes. This combined approach is particularly beneficial for patients with MCSM and associated kyphosis or localized anterior pathology that cannot be adequately managed with laminoplasty alone.

### 4.1. Clinical Relevance of MEOLP with ACF

Laminoplasty is a well-established technique for spinal cord decompression, allowing the spinal canal to expand and reducing myelopathy symptoms through posterior spinal cord repositioning [13]. However, without additional surgical intervention, laminoplasty is less effective for patients with kyphotic deformities, localized instability, or prominent anterior pathology, as these conditions limit the spinal cord’s posterior drift and may exacerbate deformity over time [11,20]. In such cases, ACF is a crucial addition to address anterior compression, correct local kyphosis, and provide stability [12]. The combined MEOLP and ACF approach leverages the advantages of both anterior and posterior decompression, providing long-term stability, functional improvement, and sustained myelopathy symptom relief.

### 4.2. Long-Term ROM Preservation

Given that ROM reduction can impact functional mobility and lead to compensatory strain on adjacent segments, ROM preservation is a key consideration in cervical spine surgeries. In our study, the ROM preservation rate was approximately 45% at 1 year postoperatively and 43% at the 9-year follow-up. Postoperative rehabilitation, including neck extension exercises, was instrumental in supporting ROM preservation, particularly during the early stages following surgery. These findings align with those of previous studies that have highlighted the importance of muscle-sparing approaches and structured rehabilitation in maintaining cervical mobility postoperatively [30].

MEOLP contributes to ROM preservation due to its muscle-sparing design. Through unilateral dissection of the paraspinal muscles, preservation of the C7 spinous process, and careful reattachment of the semispinalis cervicis muscle, MEOLP minimizes muscular disruption and fibrosis risk. This approach reduces the likelihood of postoperative axial neck pain, a common concern following standard laminoplasty [17,31]. Our study observed a reduction in VAS neck pain scores from moderate to mild that was consistent with previous research by Yoshida et al., which suggested that reduced muscle disruption mitigates the risk of axial pain and fibrosis [28], underscoring the efficacy of MEOLP as a muscle-preserving technique in enhancing patient comfort.

### 4.3. Advantages and Limitations of Anterior and Posterior Approaches

Although both anterior and posterior surgical approaches can effectively treat multilevel cervical stenosis, the sole application of the anterior approach becomes increasingly complex and risk-prone with more extensive involvement (i.e., three or more levels) [32]. High-level ACF is associated with an increased risk of complications, including dysphagia, pseudarthrosis, and, in severe cases, failure at the construct site [32,33,34]. In our cohort, ACF was limited to spinal levels with significant anterior pathology, thereby minimizing risks while providing targeted decompression. By allowing for posterior cord drift, MEOLP reduces the risk of intraoperative complications associated with extensive anterior fusion. This two-stage approach not only minimizes the chance of dural tears [35] but also addresses local kyphosis, which, if left untreated, can worsen following laminoplasty when performed without additional surgical intervention [36].

ASD is another key consideration in cervical spine surgery, because spinal segment immobilization can increase mechanical stress on adjacent levels over time, leading to degeneration. No participants in our study required reoperation for ASD, a promising outcome that may be attributed to the partial preservation of ROM at the adjacent segments. These results suggest that MEOLP with ACF not only achieved the desired decompression but also mitigated some common complications associated with more invasive, segment-immobilizing procedures [37,38].

### 4.4. Cervical Alignment and Lordotic Curvature Maintenance

Maintaining cervical alignment and lordotic curvature is essential for postoperative stability and function, particularly in patients with MCSM and preoperative kyphosis [11,39]. Loss of lordotic curvature can cause anterior spinal cord shifting, limiting the decompression achieved through posterior procedures and potentially worsening myelopathy [20]. In our study, the mean C2–C7 angle improved significantly from 8.71° ± 10.70° preoperatively to 15.23° ± 9.49° postoperatively and remained stable over the nine-year follow-up period. This suggests that MEOLP combined with ACF is effective in maintaining lordotic alignment without segmental collapse. These results align with those of Praveen et al., who emphasized the necessity of maintaining a lordotic curvature of at least 10° to optimize laminoplasty success [11]. Our findings also indicate that selecting specific levels for anterior correction based on preoperative imaging enables optimal kyphotic angle correction, contributing to the procedure’s long-term success and minimizing postoperative kyphotic changes. These observations reinforce the viability of combined MEOLP and ACF as a strategy for managing complex MCSM cases involving sagittal misalignment.

Aging has a substantial impact on cervical spine morphology, with studies indicating progressive alterations in curvature and alignment over time. Liu et al. demonstrated that aging is associated with changes in cervical spine alignment, often leading to reduced lordosis and an increased tendency toward kyphosis as degenerative processes advance [40]. These age-related changes in the cervical spine can significantly influence both clinical outcomes and surgical planning, especially when the goal is to preserve or restore sagittal alignment. Similarly, Boyle et al. highlighted the impact of aging on cervicothoracic curvature, suggesting that age-related changes affect not only cervical alignment but also the overall curvature across the cervicothoracic junction. This shift can lead to compensatory changes and altered biomechanics in the upper spine [41]. Park et al. provided normative data on cervical sagittal alignment across different age groups, emphasizing that these age-related alignment changes should be viewed as baseline variations rather than pathological deviations in asymptomatic individuals [42]. These findings underscore the importance of age-adapted surgical strategies, where cervical spine alignment goals may need to be modified for older patients to account for natural curvature changes and optimize postoperative outcomes.

### 4.5. Adjacent Segment Changes and Degeneration

Cervical spine procedures are expected to result in the gradual degeneration of adjacent segments, particularly when fusion is performed in multilevel cases [43,44]. Over time, intervertebral disks lose hydration and flexibility, potentially leading to collapse, facet hypertrophy, and spinal stenosis. Such degeneration places additional stress on unfused levels, contributing to ASD [44]. Our study observed a slight decline in adjacent-level disk height throughout the follow-up period, particularly in UDH, which significantly decreased from 6.02 ± 0.74 mm immediately after surgery to 5.82 ± 0.79 mm at 9 years. This reduction aligns with the expected natural history of cervical degeneration over time [43].

Interestingly, our multiple logistic regression results indicated UDH reduction to be a significant predictor of long-term axial neck pain. This finding indicates that progressive UDH changes may be an early indicator of ASD, highlighting the need for close monitoring of adjacent segment health during postoperative follow-up. The Pavlov ratio [28], which is used to detect cervical stenosis by comparing canal diameter to vertebral body width, showed improvement from a mean of 0.72 preoperatively to 0.90 at 1 year postoperatively, with a slight decrease to 0.87 by the 9-year follow-up. This decline, although modest, suggests that although MEOLP with ACF was shown to improve spinal canal dimensions, progressive stenotic changes in adjacent levels should be monitored to prevent potential complications.

### 4.6. Limitations, Implications, and Future Directions

Our study demonstrates that MEOLP combined with short-level ACF is a promising surgical approach for managing MCSM. However, several limitations must be acknowledged. The retrospective, single-center design limits the generalizability of our findings, as variations in patient selection, surgical techniques, and postoperative care across institutions could yield different outcomes. Although the 9-year follow-up provides valuable insights, an even longer follow-up period would be necessary to fully assess the durability of surgical outcomes and the progression of ASD. Furthermore, our reliance on radiographic and functional measures, such as ROM and cervical alignment, restricts the scope of patient evaluation. Incorporating quality-of-life assessments and patient-reported outcomes, such as satisfaction and daily functionality, would provide a more comprehensive view of surgical success. Lastly, the absence of a control group prevents causal inferences, and while statistical adjustments were made, confounding variables such as age and baseline severity of myelopathy remain potential sources of bias. The clinical implications of this study highlight the benefits of combining MEOLP with short-level ACF, particularly for complex cases involving kyphosis or significant anterior pathology. The combined approach effectively addresses both posterior and anterior spinal compression while maintaining alignment and preserving functional mobility. The muscle-sparing design of MEOLP contributes to reduced axial neck pain and greater ROM preservation compared to standard laminoplasty. These attributes make the procedure particularly valuable for addressing the multifaceted challenges of MCSM.

Our study still had some limitations. First, conducting multi-center, prospective cohort studies or randomized controlled trials would improve the external validity of the findings and minimize selection bias. Second, incorporating standardized patient-reported outcomes, such as the Neck Disability Index or EQ-5D, would offer a more holistic view of patient recovery and satisfaction. Third, extending the follow-up period beyond 9 years could help clarify the trajectory of ASD and its impact on cervical alignment and function. Fourth, developing control groups matched by age, severity of myelopathy, and preoperative alignment would enable a more precise evaluation of the efficacy of MEOLP combined with ACF. Finally, biomechanical analyses focusing on stress distribution on adjacent segments could guide strategies to mitigate ASD and improve surgical techniques. Despite its limitations, this study provides critical insights into the long-term effectiveness of MEOLP combined with short-level ACF for MCSM. Addressing these limitations and expanding the scope of future research to include advanced imaging modalities, optimized rehabilitation protocols, and patient-centered metrics will be pivotal in refining surgical strategies for complex spinal disorders and improving patient outcomes.

## 5. Conclusions

Our study demonstrated that MEOLP combined with short-level ACF can offer a durable and effective approach for managing MCSM, achieving substantial decompression, alignment correction, and ROM preservation over a 9-year follow-up period. This combined approach addressed both anterior and posterior pathologies, providing a reliable solution for complex cases with kyphosis or anterior compression, in which traditional laminoplasty alone may be insufficient. Although ASD remains a potential complication, especially for patients with preoperative disk height alterations, integrating protective strategies such as tailored physiotherapy may further improve surgical outcomes. Overall, our findings suggest that MEOLP with ACF is a viable treatment option for MCSM, paving the way for refined management strategies that optimize both functional outcomes and quality of life for patients.

## Figures and Tables

**Table 1:** Demographics (*n* = 124).

Item	Male	Female	Total	*p*-Value
N	64	60	124	
Age	55 ± 11.89	58.53 ± 11.71	56.71 ± 11.75	0.412
Myelomalacia (%)	22 (68.8%)	12 (40.0%)	34 (54.8%)	0.108
Baseline				
Pavlov ratio (MACL-2 level)	0.71 ± 0.05	0.72 ± 0.04	0.72 ± 0.05	0.586
C2–C7 angle	−8.88 ± 10.02	−8.53 ± 11.73	−8.71 ± 10.70	0.931
CSVA	18.57 ± 10.24	12.60 ± 8.83	15.68 ± 9.90	0.094
C7 slope	16.19 ± 6.78	13.80 ± 4.21	15.03 ± 5.72	0.253
ACF angle	−2.69 ± 6.91	−3.80 ± 6.54	−3.23 ± 6.64	0.649
ACF height	54.78 ± 10.85	52.37 ± 11.47	53.61 ± 11.03	0.552
UDH	6.47 ± 0.84	5.94 ± 0.53	6.21 ± 0.75	0.049 *
LDH	6.33 ± 0.83	5.90 ± 1.01	6.12 ± 0.93	0.208
ROM	48.73 ± 14.52	44.47 ± 12.77	46.60 ± 13.61	0.400
UROM	17.19 ± 9.09	17.60 ± 11.36	17.39 ± 10.08	0.912
LROM	10.06 ± 12.64	8.60 ± 8.18	9.35 ± 10.57	0.707
JOA	10.56 ± 1.26	11.40 ± 0.74	10.97 ± 1.11	0.033 *
Neck VAS	6.63 ± 0.62	6.40 ± 0.99	6.52 ± 0.81	0.450

Data are presented as *n* or mean ± stdev.* *p*-value < 0.05 was considered statistically significant after the test. CSVA, C2–C7 sagittal vertical axis; ACF, anterior cervical fusion; UDH, upper level disk height; LDH, lower level disk height; ROM, range of motion; UROM, upper-level range of motion; LROM, lower-level range of motion; JOA, Japanese Orthopedic Association; Neck VAS, neck visual analog scale; MACL-2, the most affected cervical level minus two.

**Table 2:** Comparison of the change in different parameters (*n* = 124).

Item	Pre-OP	Post-OP			Post-OP 9Y		
		Value	Diff.	*p*-Value	Value	Diff.	*p*-Value
Pavlov ratio (MACL-2 level)	0.72 ± 0.05	0.90 ± 0.04	0.19 ± 0.05	<0.001 *	0.87 ± 0.04	0.16 ± 0.06	<0.001 *
C2–C7 angle	−8.71 ± 10.70	−15.23 ± 9.49	−6.52 ± 8.68	<0.001 *	−14.90 ± 9.33	−6.19 ± 8.85	0.001 *
CSVA	15.68 ± 9.90	24.04 ± 11.11	8.36 ± 12.32	0.001 *	25.07 ± 12.90	9.39 ± 11.86	<0.001 *
C7 slope	15.03 ± 5.72	20.06 ± 7.07	5.03 ± 5.50	<0.001 *	22.45 ± 7.21	7.42 ± 6.37	<0.001 *
ACF angle	−3.23 ± 6.64	−14.10 ± 9.30	−10.87 ± 11.54	<0.001 *	−13.61 ± 8.79	−10.39 ± 10.03	<0.001 *
ACF height	53.61 ± 11.03	58.45 ± 11.71	4.84 ± 3.21	<0.001 *	56.35 ± 10.34	2.73 ± 3.66	<0.001 *
UDH	6.21 ± 0.75	6.02 ± 0.74	−0.19 ± 0.51	0.044 *	5.82 ± 0.79	−0.39 ± 0.68	0.003 *
LDH	6.12 ± 0.93	6.05 ± 0.99	−0.08 ± 0.42	0.315	5.54 ± 1.10	−0.58 ± 0.73	<0.001 *
ROM	46.60 ± 13.61	20.80 ± 6.88	−25.80 ±1 3.12	<0.001 *	19.83 ± 8.70	−26.77 ± 14.54	<0.001 *
UROM	17.39 ± 10.08	13.58 ± 7.02	−3.81 ± 8.25	0.015 *	13.58 ± 9.41	−3.81 ± 10.50	0.053
LROM	9.35 ± 10.57	6.23 ± 7.46	−3.13 ± 6.40	0.011 *	4.35 ± 5.04	−5.00 ± 8.87	0.004 *
JOA	10.97 ± 1.11	15.52 ± 0.68	4.55 ± 0.96	<0.001 *	16.10 ± 0.94	5.13 ± 1.28	<0.001 *
Neck VAS	6.52 ± 0.81	3.00 ± 0.82	−3.52 ± 0.93	<0.001 *	3.16 ± 1.34	−3.35 ± 1.52	<0.001 *

Data are presented as mean ± stdev. * *p*-value < 0.05 was considered statistically significant after the test. CSVA, C2–C7 sagittal vertical axis; ACF, anterior cervical fusion; UDH, upper level disk height; LDH, lower level disk height; ROM, range of motion; UROM, upper-level range of motion; LROM, lower-level range of motion; JOA, Japanese Orthopedic Association; Neck VAS, neck visual analog scale; MACL-2, the most affected cervical level minus two.

**Table 3:** The risk factors associated with post-op 9Y change in JOA and Neck VAS (*n* = 124).

Item	Diff. in JOA		Diff. in Neck VAS	
	β (95% CI)	*p*-Value	β (95% CI)	*p*-Value
Age	0.05 (−0.01, 0.10)	0.105	−0.05 (−0.11, 0.01)	0.087
Sex (M vs. F)	0.37 (−0.80, 1.55)	0.515	0.95 (−0.24, 2.15)	0.113
Myelomalacia (Y vs. N)	0.14 (−1.03, 1.32)	0.800	−0.8 (−1.99, 0.39)	0.178
Pavlov ratio (Pre) (MACL-2 level)	−1.95 (−14.24, 10.34)	0.744	−0.19 (−12.7, 12.32)	0.975
Diff. in UDH	−0.23 (−1.41, 0.95)	0.685	1.46 (0.26, 2.66)	0.020 *
Diff. in LDH	0.03 (−1.35, 1.41)	0.963	−0.26 (−1.67, 1.14)	0.699
Diff. in ROM	−0.05 (−0.12, 0.01)	0.116	0.10 (0.03, 0.17)	0.009 *
Diff. in UROM	0.04 (−0.04, 0.12)	0.336	−0.08 (−0.16, 0.01)	0.069
Diff. in LROM	0.08 (−0.03, 0.19)	0.143	−0.11 (−0.22, −0.001)	0.049 *

Data are presented as β (95% CI). * *p*-value < 0.05 was considered statistically significant after the test. JOA, Japanese Orthopedic Association; Neck VAS, neck visual analog scale; UDH, upper level disk height; LDH, lower level disk height; ROM, range of motion; UROM, upper-level range of motion; LROM, lower-level range of motion; JOA, Japanese Orthopedic Association; Neck VAS, neck visual analog scale; MACL-2, the most affected cervical level minus two.

## Data Availability

Data are contained within the article.
